# Reliability and validity of the Cancer Therapy Satisfaction Questionnaire in lung cancer

**DOI:** 10.1007/s11136-015-1062-z

**Published:** 2015-07-21

**Authors:** K. Cheung, M. de Mol, S. Visser, B. L. Den Oudsten, B. H. Stricker, J. G. J. V. Aerts

**Affiliations:** Department of Epidemiology, Erasmus MC - University Medical Centre Rotterdam, P.O. Box 2040, 3000 CA Rotterdam, The Netherlands; Department of Pulmonary Diseases, Amphia Hospital Breda, Breda, The Netherlands; Department of Pulmonary Diseases, Erasmus MC - University Medical Centre Rotterdam, Rotterdam, The Netherlands; Inspectorate of Health Care, The Hague, The Netherlands; Department of Medical and Clinical Psychology, Centre of Research on Psychology in Somatic Diseases (CoRPS), Tilburg University, Tilburg, The Netherlands

**Keywords:** Cancer Therapy Satisfaction Questionnaire, Lung cancer, Chemotherapy, Satisfaction, Expectations, Validity

## Abstract

**Purpose:**

To test the reliability and validity of the Cancer Treatment Satisfaction Questionnaire (CTSQ), to assess its relation with quality of life (QoL), and to assess the interpretability of the domain scores in lung cancer patients receiving intravenous chemotherapy.

**Methods:**

Patients with stage IIIB and IV non-squamous non-small cell lung carcinoma treated with pemetrexed were enrolled in our study. They completed the 16-item CTSQ and two other (health-related) QoL questionnaires. Information about sociodemographic characteristics, cancer stage, and the experience of adverse events was collected. Internal consistency, construct validity, and clinical interpretability were calculated.

**Results:**

Fifty-five patients completed the CTSQ. Correlations of the CTSQ items with its domain were all above 0.40. A high correlation between item 8 and the expectations of therapy and satisfaction with therapy domain was observed (0.50 and 0.48, respectively). The CTSQ domains demonstrated good internal consistency and low to moderate correlations of the CTSQ with the European Organization for Research and Treatment of Cancer Quality of Life Questionnaire-C30 and World Health Organization Quality of Life-BREF. No significant differences in mean domain scores were observed in relation to the number and severity of different adverse events and chemotherapy-related adverse events.

**Conclusions:**

The Dutch version of the CTSQ was found to be a reliable and valid instrument to assess satisfaction and expectations of treatment in lung cancer patients receiving intravenous chemotherapy. Furthermore, the CTSQ proved to be of additional informative value as not all of its domains correlated with the various domains of the existing HRQoL instruments.

## Introduction

Anticancer therapies mostly offer modest improvements in survival, making the occurrence of adverse events an important outcome parameter in studies and clinical practice. It is well established that adverse events impair health-related quality of life (HRQoL) [1] and that (change of) HRQoL acts as a prognostic factor in (lung) cancer patients [2–7]. Questionnaires evaluating HRQoL offer valuable information about the impact of cancer and therapy-related adverse events. However, they do not address patients’ satisfaction, expectations, and preferences concerning the occurrence and management of adverse events, the choice and type of therapy, and the efficacy of treatment. Such information provides opportunities for physicians to improve therapy compliance, personalize the course of treatment, and develop interventions designed to prevent or effectively treat adverse events and thus improve HRQoL. Certainly in diseases with a poor prognosis (e.g., advanced lung cancer) where the treatment is associated with only limited increases in survival and elevated risks for adverse events, insight into patients’ expectations and satisfaction is of upmost importance.

In 2005, the CTSQ was developed to assess patients’ opinions and feelings concerning their cancer therapy and associated adverse events [8]. A psychometric validation study of this questionnaire was performed, which resulted in an optimized and more brief version ensuring its reliability for research purposes [9]. Since then, the CTSQ has only been validated in a Korean study in which just four patients were treated with chemotherapy [10].

Given these considerations, the objective of our study was focused on three main aspects of the CTSQ: (1) to test the reliability and validity of the Cancer Treatment Satisfaction Questionnaire (CTSQ) in patients with lung cancer receiving intravenous chemotherapy, (2) to assess its relation to health-related quality of life (HRQoL), and (3) to assess the interpretability of the domain scores.

## Materials and methods

### Study population

This study was approved by the Institutional Review Board of the Erasmus University Medical Center in Rotterdam, the Netherlands. Patients were recruited from a university hospital (Erasmus University Medical Center) and a large teaching hospital (Amphia hospital) specialized in lung cancer care located in the western part of the Netherlands. Patients were enrolled in our study if they met the following criteria: They provided written informed consent, were aged 18 years or older, and were treated with at least four cycles of pemetrexed monotherapy or in combination with cisplatin or carboplatin as either first or second line. Patients were excluded if they met the following criteria: They were not able to read Dutch or could not complete the questionnaire because of a physical or mental condition (which prohibited participation in the study). A sample size of at least 50 patients was needed in order to perform a validation study [11].

### Study measures

The CTSQ contains three domains covering 16 items: expectations of therapy (ET; five items), feelings about side effects (FSE; four items) and satisfaction with therapy (SWT; seven items). Each item was scored on a scale from one to five with a value of one corresponding with the worst response and a value of five representing the best response. Four items are reverse-coded. Domain score was calculated by the formula: (mean of completed item scores −1) × 25. This results in a domain score ranging from 0 to 100, with a higher score representing a better outcome on each domain.

The original CTSQ was translated into Dutch by TransPerfect Translations Inc. according to the forward/backward methodology following international guidelines [12]. Questions were translated in a forward manner (English to Dutch) by two independent native-speaking linguists of the target language experienced in the translation of quality of life instruments. A third independent native speaker reviewed these translations and selected the most appropriate translation of the items or provided an alternative version. Discrepancies, linguistic limitations or cultural differences were addressed. Back translation was performed by a fourth independent native speaker with proficiency in English. An oncologist determined whether the Dutch translation was in line with the medical terminology as used in the Netherlands. Finally, five respondents who received cancer treatment in the past 18 months asked to provide feedback on the Dutch CTSQ during an interview. The respondents’ overall impression of the instrument was that it was “easy to complete.” The respondents’ answers corresponded with the intended meanings of the items. During the translation process, some questions were slightly changed (i.e., not literally translated) to ensure conceptual equivalence and cultural relevance to facilitate correct use of Dutch grammar. Permission of use was granted by Pfizer Inc. the current owner of the intellectual rights of the CTSQ. A pre-assessment of the Dutch version was conducted in 14 patients with lung cancer (not included in this study) to assess whether the questions were understandable and acceptable for use in the study.

The European Organization for Research and Treatment of Cancer Quality of Life Questionnaire-Core 30 (EORTC QLQ-C30) is a cancer-specific HRQoL instrument with demonstrated psychometric properties [13]. It consists of 30 items and incorporates a global health status/quality of life scale, five functional scales and a number of single items assessing additional symptoms or difficulties. Each of the QLQ-C30 domains is scored on a 0–100 scale, with higher scores on the functional scales being indicative of better HRQoL, whereas higher scores on the symptom scales are reflective of worse symptoms [14, 15].

The World Health Organization Quality of Life-BREF (WHOQOL-BREF) is a shorter version of the original WHOQoL-100 questionnaire. It is a generic QoL instrument and comprises 26 items divided over 4 domains: physical health, psychological health, social relationships, and environment and one facet: overall quality of life and general health. The WHOQOL-BREF domains are scored on a 4–20 scale and the facet on a 2–10 scale with higher scores indicating a better quality of life [16]. The WHOQOL-BREF is a well-established instrument that was developed for use in a wide range of disease areas and health problems [17].

All questionnaires were completed after patients finished their four-cycle therapy of chemotherapy. In addition to completing the instruments, respondents were asked to provide information about the frequency and severity of adverse events they have experienced (cancer or therapy related). We also collected sociodemographic information (age, gender, educational level, ethnicity, smoking status, and clinical history) and information about cancer stage, hospitalization (due to cancer or adverse effect of therapy), and the ECOG performance status.

### Statistical analysis

Floor and ceiling effects were calculated in our study and were considered to be present if more than 15 % of the respondents achieved the lowest (floor effect) or highest (ceiling effect) possible domain score [11].

Construct validity was evaluated using Pearson’s rank correlation coefficient between questionnaire items and domains. Correlations of 0.40 or higher indicate a good correlation between items and domains [11].

Internal consistency reliability measures to which extent items within a domain correlate with each other to form a (multi-item) domain. Reliability coefficients for the CTSQ domains were estimated using Cronbach’s coefficient alpha where a reliability coefficient of 0.70 or higher was considered to be acceptable [11].

Known-groups validity comparisons were made for the CTSQ domains in relation to the number of different adverse events and its severity. Also the impact of therapy-related adverse events compared to cancer-related adverse events on CTSQ domain score was evaluated. For this analysis, the one-way ANOVA was used to determine whether there are any significant differences between the means of two or more independent groups.

The association between the CTSQ domains with domains of the EORTC QLQ-C30 and WHOQOL-BREF was assessed using Spearman’s correlation coefficients.

We assessed interpretability, which is defined as the degree to which one can assign qualitative meaning to quantitative scores. For each CTSQ domain, the MCID was calculated using the approach of 0.5 SD [18] and 1 standard error of measure (SEM) [19–21]. MCID is the smallest change in an outcome that a patient would identify as important. The 0.5 SD benchmark of an outcome measure entails that patients improving more than 0.5 of the outcome score’s SD have achieved a minimally clinically important difference [22]. For the 1 SEM approach, we have used the internal consistency reliability estimates. In addition, results of the known-groups comparison were used to derive the MCID using the number of adverse events with Common Terminology Criteria for Adverse Events (CTCAE) grade 3 or 4 as an anchor. A *p* value below 0.05 was considered to be statistically significant.

All analyses were performed using SPSS version 21.0 (IBM Corporation, Armonk, NY).

## Results

### Patient characteristics

Table 1 describes the characteristics of our study population. A total of 55 patients completed the questionnaires. The age of these patients ranged from 45 to 79 years, with a mean of 55.0 (SD 8.6). Forty-four patients indicated they had received a low level of education (80.0 %), and 32.7 % stated to be employed. The majority of these patients were diagnosed with adenocarcinoma of the lung (94.5 %), and 85.5 % had stage IV NSCLC. Almost all patients (98.2 %) had a good ECOG performance score (grade 0 or 1). The majority of patients received pemetrexed chemotherapy as a first-line treatment (85.5 %).

**Table 1:** Characteristics of study population

Characteristic	Overall sample (*n* = 55)
Age, years	
Mean (SD)	55 (8.6)
Min, max	45, 79
Sex	
Male	27 (49.1)
Ethnicity	
White/Caucasian	52 (94.5)
Asian	1 (1.8)
Negroid	1 (1.8)
Other	1 (1.8)
Education^a^	
Low	44 (80.0)
High	8 (14.5)
Unknown	3 (5.5)
Employment	
Yes	18 (32.7)
Marital status	
Married/cohabiting	44 (80.0)
Unmarried partners/not cohabiting	3 (5.5)
Divorced/separated	2 (3.6)
Widowed/partner died	4 (7.3)
Single	1 (1.8)
Unknown	1 (1.8)
Cancer stage^b^	
Locally advanced (IIIB)	4 (7.3)
Metastatic (IV)	47 (85.5)
Other	4 (7.3)
Type of tumor^b^	
Adenocarcinoma	52 (94.5)
Large cell carcinoma	1 (1.8)
Mesothelioma	1 (1.8)
Large cell neuroendocrine carcinoma	1 (1.8)
Line of therapy	
First line	47 (85.5)
Second line	5 (9.1)
Adjuvant	3 (5.5)
ECOG performance status^a^	
Grade 0	17 (30.9)
Grade 1	38 (69.1)

Values are given in numbers (percentages) unless stated otherwise

*n* number of patients, *SD* standard deviation, *ECOG* Eastern Cooperative Oncology Group (ECOG)

^a^Low education: persons whose highest level of education is primary education, lower general education or lower vocational education. High education: persons whose highest level of education is higher general education, higher vocational education or university

^b^Measured at baseline

### Mean scores and floor and ceiling effects

The mean scores of the ET and FSE domain were 55.6 (SD 22.5) and 52.2 (SD 23.8), respectively. The SWT domain had a mean score of 79.7 (SD 13.9), which was much higher compared to the mean scores of the other domains. No patients demonstrated the lowest possible domain score of 0.0. The floor effects for all domains were therefore zero. The FSE domain did not reach the highest possible score of 100, resulting in a negligible ceiling effect for this domain. For the ET and SWT domain, we observed a ceiling effect of 5.5 and 9.1 % respectively, which is below the common accepted limit of 15 % (Table 2).

**Table 2:** Summary statistics for CTSQ domains

CTSQ domain	*n*	Mean (SD)	Median	Observed range (min, max)	Floor effect *n* (%)	Ceiling effect *n* (%)
Expectations of therapy (ET)	55	55.6 (22.5)	55.0	15.0, 100.0	0 (0.0)	3 (5.5)
Feelings about side effects (FSE)	54	52.2 (23.8)	56.3	12.5, 93.8	0 (0.0)	0 (0.0)
Satisfaction with therapy (SWT)	55	79.7 (13.9)	82.1	42.9, 100.0	0 (0.0)	5 (9.1)

*SD* standard deviation, *n* number of patients, *CTSQ* Cancer Therapy Satisfaction Questionnaire

### Construct validity

Construct validity was supported for all 16 items as we observed a correlation of 0.40 or higher with their own hypothesized domain. However, we found that item 8 (chemotherapy would help you live longer) had a similar correlation with ET domain (0.50) as the SWT domain (0.48). All other comparisons showed good results, as these items correlated better with their own hypothesized domain than with competing domains (Table 3).

**Table 3:** Construct validity of the CTSQ (*n* = 55)

Item number	Description	ET correlation coefficient (sig.)	FSE correlation coefficient (sig.)	SWT correlation coefficient (sig.)
Expectations of therapy (ET)				
1	CT would help you to return to a normal life	**0.73** (<0.001)	−0.20 (0.16)	−0.04 (0.77)
2	CT would get rid of the cancer	**0.87** (<0.001)	0.07 (0.61)	−0.006 (0.97)
3	CT would help prevent the cancer from coming back	**0.89** (<0.001)	0.13 (0.33)	0.20 (0.15)
4	CT would stop the cancer from spreading	**0.81** (<0.001)	−0.04 (0.80)	0.34 (0.01)
8	CT would help you live longer	**0.50** (<0.001)	0.15 (0.39)	**0.48** (<0.001)
Feelings about side effects (FSE)				
5R^a^	CT limited your daily activities	0.002 (0.99)	**0.68** (<0.001)	0.23 (0.09)
6R^a^	Upset about side effects	0.02 (0.91)	**0.80** (<0.001)	0.14 (0.30)
11R^a^	Overall, was taking CT as difficult as expected	−0.05 (0.70)	**0.91** (<0.001)	0.20 (0.14)
13	Overall, were side effects as expected	0.12 (0.38)	**0.87** (<0.001)	**0.41** (0.002)
Satisfaction with therapy (SWT)				
7	CT was worth taking even with side effects	0.37 (0.006)	0.08 (0.56)	**0.70** (<0.001)
9R^a^	How often did you think about stopping CT	−0.08 (0.56)	0.30 (0.03)	**0.42** (0.002)
10	Overall, how worthwhile was your CT	0.29 (0.03)	0.02 (0.89)	**0.63** (<0.001)
12	Overall, how well did the benefits of CT meet your expectations	0.27 (0.05)	0.25 (0.06)	**0.79** (<0.001)
14	How satisfied were you with the form of your CT	−0.11 (0.45)	0.19 (0.17)	**0.57** (<0.001)
15	How satisfied were you with your most recent CT	0.09 (0.51)	**0.40** (0.003)	**0.64** (<0.001)
16	If given choice again, would you decide to take this CT treatment	0.02 (0.87)	0.28 (0.04)	**0.74** (<0.001)

Correlations of CTSQ domains with CTSQ items of 0.40 or larger are in bold

*sig.* significance (2-tailed), *CT* chemotherapy, *CTSQ* Cancer Therapy Satisfaction Questionnaire

^a^These items were reverse-coded by subtracting the original value from 6, where a value of 1 represents the worst response and a value of 5 represents the best response

### Internal consistency

The internal consistency of the CTSQ domains is given in Table 4. All three domains met the reliability coefficient of 0.70 or higher. Cronbach’s alpha of the ET and FSE domains were both above 0.80 (0.83), except for the SWT domain (0.77). As presented in Table 3, we observed that item 8 had a similar correlation with the SWT domain as with the ET domain. We explored whether moving item 8 from the ET domain to the SWT domain would improve Cronbach’s alpha for both the ET and SWT domains. We found a slight increase in the alpha coefficients of both domains (ET: 0.86, SWT: 0.79).

**Table 4:** Internal consistency of CTSQ domains

CTSQ domain	Internal consistency	Internal consistency (revised)
	Cronbach’s alpha	Cronbach’s alpha^a^
	*n* = 55	*n* = 55
Expectations of therapy (ET)	0.83	0.86
Feelings about side effects (FSE)	0.83	0.83
Satisfaction with therapy (SWT)	0.77	0.79

*n* number of patients who completed the CTSQ questionnaire, *CTSQ* Cancer Therapy Satisfaction Questionnaire

^a^Item 8 was moved from the ET domain to the SWT domain

### Known-groups comparisons

Table 5 shows the known-groups validity comparisons for the CTSQ domains in relation to the number of different adverse events, its severity and chemo-related adverse events. None of these results were found to be significant. We observed an increasing number of grade 3 and 4 adverse events that corresponded with a decreasing mean score of the FSE domain. The same observation was found in the analysis where we looked at the percentage of adverse events that were related to chemotherapy. Also, frequency and severity of adverse events were not related to satisfaction with therapy.

**Table 5:** Known-groups comparisons (n = 55)

Description	CTSQ expectations of therapy			CTSQ feelings about side effects			CTSQ satisfaction with therapy		
	*n*	Mean (SD)	*P* value (effect size)*	*n*	Mean (SD)	*P* value (effect size)*	*n*	Mean (SD)	*P* value (effect size)*
Number of different adverse events^a^									
0–10	27	56.2 (24.7)	0.86	26	55.3 (22.9)	0.36	27	79.1 (13.2)	0.77
More than 10	28	55.1 (20.6)		28	49.3 (24.7)		28	80.2 (14.7)	
Number of adverse events with CTCAE grade 3 or 4^a^									
0	25	57.1 (22.7)	0.17	24	53.6 (23.6)	0.41	25	77.5 (14.4)	0.47
1	10	42.3 (16.3)		10	51.9 (23.0)		10	80.0 (14.4)	
2 or 3	12	63.3 (27.2)		12	57.8 (26.1)		12	85.1 (11.0)	
More than 3	8	56.3 (16.4)		8	39.8 (21.6)		8	77.7 (15.8)	
Percentage of adverse events that are related to chemotherapy									
0–25	6	63.3 (23.2)	0.35	6	56.3 (22.7)	0.56	6	84.5 (14.0)	0.65
26–50	11	61.6 (23.8)		10	55.0 (22.6)		11	76.0 (9.5)	
51–75	23	49.5 (21.4)		23	54.9 (25.7)		23	80.7 (14.1)	
76–100	15	57.7 (22.6)		15	44.6 (22.5)		15	78.8 (16.5)	

* Effect sizes were only shown where one-way ANOVA was significant (*P* < 0.05)

*CTSQ* Cancer Therapy Satisfaction Questionnaire, *SD* standard deviation, *n* number of patients who completed the questionnaire, *CTCAE* Common Terminology Criteria for Adverse Events

^a^Reported adverse events: 2 weeks prior to last chemo until 4 weeks after last chemo

### Minimally clinically important differences

The estimates of the MCIDs are given in Table 6. Estimates of the MCID for the ET and FSE domain were almost the same (0.5 SD: 11.75; 1 SEM: 9.69 and 0.5 SD: 12.4; 1 SEM: 9.28, respectively). The calculated estimates using the 0.5 SD approach were higher for both domains compared to the estimates using the 1 SEM approach. We observed a much lower estimate for the SWT domain (0.5 SD: 6.55; 1 SEM: 6.14) and a smaller difference between the estimates of the 0.5 SD and 1 SEM. The anchor-based MCID was estimated by calculating the average change in CTSQ score. For the ET domain, the estimate that was obtained using the number of grade 3 or 4 adverse events as an anchor was higher than the observed estimates using the 0.5 SD and 1 SEM approach (14.3). For the other two domains, we observed lower estimates when using the anchor-based method (SE: 8.5 and SWT: 5). However, results that were obtained using this method need to be interpreted carefully as the effect size could not be measured due to the low numbers of patients.

**Table 6:** Estimates of minimally clinically important differences on CTSQ domains

CTSQ domain	0.5 SD^a^	1 SEM^b^	Known-groups differences^c^
Expectations of therapy	11.25	9.28	A difference of 14.8 points between 0 and 1 AE, 21 points difference between 1 and 2/3 AEs and a difference of 7 points between 2/3 and >3 AEs. The average difference is 14.3 points
Feelings about side effects	11.9	9.81	A difference of 1.7 points between 0 and 1 AE, 5.9 points difference between 1 and 2/3 AEs and a difference of 18 points between 2/3 and >3 AEs. The average difference is 8.5 points
Satisfaction with therapy	6.95	6.37	A difference of 2.5 points between 0 and 1 AE, 5.1 points difference between 1 and 2/3 AEs and a difference of 7.4 points between 2/3 and >3 AEs. The average difference is 5 points

*n* number of patients who completed the CTSQ questionnaire, *CTSQ* Cancer Therapy Satisfaction Questionnaire, *CTCAE* Common Terminology Criteria for Adverse Events, *SD* standard deviation, *SEM* standard error of measure

^a^0.5 SD of CTSQ domain scores

^b^Using internal consistency reliability estimates

^c^Using the known-group criterion ‘number of adverse events with CTCAE grade 3 or 4′

### Correlation of CTSQ domains with quality of life

The correlation between the CTSQ domains and domains of the EORTC QLQ-C30 is shown in Table 7. We found the FSE domain correlated more strongly with the EORTC QLQ-C30 domains than the other two CTSQ domains. The highest correlations (*r* ≥ 0.40) were observed with global health status, role functioning, emotional functioning and the symptom domains fatigue, nausea and vomiting, and appetite loss. No correlation of 0.40 or higher was observed between the ET domain and the HRQoL domains. The SWT domain only significantly correlated with nausea and vomiting (*r* = −0.41). The negative correlations between the CTSQ and HRQoL domains indicate that higher scores of the CTSQ domains are associated with worse symptoms.

**Table 7:** Correlations of CTSQ with EORTC QLQ-C30 domains

*n* = 55	CTSQ domains		
	Expectations of therapy	Feelings about side effects	Satisfaction with therapy
EORTC QLQ-C30 domains			
Global health status/quality of life	0.01	**0.40** ^b^	0.27^a^
Physical functioning	0.18	0.34^a^	0.20
Role functioning	0.13	**0.48** ^b^	0.09
Emotional functioning	−0.011	**0.51** ^b^	0.17
Cognitive functioning	0.006	0.18	−0.03
Social functioning	−0.080	0.32^a^	0.02
Fatigue	−0.10	−**0.52** ^b^	−0.22
Nausea and vomiting	−0.04	−**0.53** ^b^	−**0.41** ^b^
Pain	−0.006	−0.26	−0.17
Dyspnea	0.018	−0.23	0.07
Insomnia	−0.16	0.10	−0.06
Appetite loss	−0.07	−**0.60** ^b^	−0.30^a^
Constipation	−0.20	−0.39^b^	−0.11
Diarrhea	−0.15	−0.11	0.04
Financial difficulties	−0.09	−0.04	0.04

Spearman’s correlations. Correlations of CTSQ domains with EORTC QLQ-C30 domains of *r* ≥ 0.40 or larger are in bold

*CTSQ* Cancer Therapy Satisfaction Questionnaire, *EORTC QLQ-C30* European Organization for Research and Treatment of Cancer Quality of Life Questionnaire-Core 30, *n* number of patients who completed the questionnaire

^a^Correlation is significant at the 0.05 level (2-tailed)

^b^Correlation is significant at the 0.01 level (2-tailed)

Results of the association between the CTSQ and WHOQOL-BREF domains are presented in Table 8. The domains of WHOQOL-BREF had the strongest correlations with the FSE domain. However, only the psychological domain had a correlation above 0.40 (*r* = 0.52).

**Table 8:** Correlations of CTSQ with WHOQOL-BREF domains

*n* = 55	CTSQ domains		
	Expectations of therapy	Feelings about side effects	Satisfaction with therapy
WHOQOL-BREF domains			
Overall quality of life and general health	0.20	0.28^a^	0.14
Physical health	0.10	0.36^b^	0.10
Psychological health	0.21	**0.52** ^b^	0.24
Social relationships	0.07	0.12	0.12
Environment	0.04	0.15	0.04

Spearman’s correlations. Correlations of CTSQ domains with WHOQOL-BREF domains of *r* ≥ 0.40 or larger are in bold

*CTSQ* Cancer Therapy Satisfaction Questionnaire; *WHOQoL-Bref* World Health Organization Quality of Life-Bref, *n* number of patients who completed the questionnaire

^a^Correlation is significant at the 0.05 level (2-tailed)

^b^Correlation is significant at the 0.01 level (2-tailed)

## Discussion

Although HRQoL questionnaires inform healthcare professionals about the well-being of their patients, they do not address patients’ expectations and satisfaction with therapy. Brown et al. [1] demonstrated that expectations of therapy and adverse events are important determinants for patient compliance. In addition, satisfaction is likely to express contentment with therapy and may also be influenced by the occurrence of adverse events. The CTSQ could be used as a tool to monitor the management of therapy and adverse events to improve HRQoL. Especially in cancer patients with a limited prognosis, this may be of importance. Therefore, our objective was to evaluate the reliability and validity of the CTSQ. Our study showed good results and hence supports the construct validity and internal consistency reliability of the CTSQ.

The previous psychometric validation study demonstrated a positively skewed score distribution of the ET domain with a substantial ceiling effect (20.5) [9]. Even higher ceiling effects were observed in the study by Park et al. for the ET and FSE domains (21.6 and 36.3, respectively) [9]. No floor or ceiling effects were found in our study, which indicates that no extreme items are missing in the lower or upper end of the scale. This might be explained by the fact that all patients in our study had advanced stage lung cancer of whom all have a limited survival compared to those with a curable disease. As lung cancer patients in general demonstrate information-seeking behavior to cope with their disease [23] and the patients in our study were already informed about their limited survival prior to the start of therapy, we assume that the patients enrolled in our study did not have such high expectations. Moreover, disease stage may also influence the FSE and SWT domains. Simultaneously with disease progression, patients may experience more and severe cancer-related adverse events. These adverse events may be attributed by patients to chemotherapy probably resulting in a lower FSE domain score and decreased satisfaction with therapy.

All items correlated better with their own domains than with the other domains, which is in line with the results of the psychometric validation study. However, the correlations between the items and domains were found to be higher in our study compared with the previous study, which might be explained by the homogeneity of the population in our study. We observed that item 8 of the CTSQ (cancer therapy would help you live longer) had strong correlations with the SWT domain and with its own ET domain, which is in line with the results of the previous CTSQ studies [9, 10]. Moreover, when we moved item 8 from the ET to the SWT domain, it resulted in a slight increase in alpha coefficients for both the ET and SWT domains. However, the sample size in our study was small. Therefore, we suggest further research to be conducted in a larger population to confirm this finding.

In 2004, a validation study of another patient satisfaction questionnaire (TSQM) was performed and showed significant differences in patient satisfaction and convenience of treatment between different treatment modalities (e.g., oral, topical, injectable, inhaler) [24]. As patients in our study received only intravenously administered chemotherapy, we expect this may have affected the generalizability of our results. In addition, all patients in our study were diagnosed with advanced lung cancer, whereas patients with various diseases were included in the TSQM validation study [24]. This may also hamper broad application of the CTSQ. However, when we compare our study with the study of Trask et al., which was conducted in a more heterogeneous population, we observed similar results with respect to construct validity and internal consistency reliability. Therefore, we assume that the single route of administration and the disease stage of the included patients in our study did not have a major impact on our results in terms of generalizability.

As for the estimates of the MCIDs using the distribution-based methods, we observed similar results for the FSE and SWT domains when we compare our results (FSE 11.9, 9.81; SWT 6.95, 6.37) with the results of the previous psychometric validation study (FSE 11.0, 10.55; SWT 6.88, 5.84). However, we found a clear difference of the MCIDs of the ET domain between both studies as in our study a larger change of domain score is needed for it to be considered clinically relevant (MCIDs in our study: 11.25, 9.28; Trask et al. [9]: 9.59, 6.92). A possible explanation for this is the ceiling effect of 20.5 %, which was observed in the study by Trask, which was not observed in our study. Consequently, they were not able to detect such a difference, because this change would then exceed the range of the scale.

We observed an increasing number of severe and chemotherapy-related adverse events that corresponded with a decreasing mean FSE domain score. According to Grutters et al. [25], this may be due to the impact of adverse events on HRQoL as they showed in their study that already moderate adverse events resulted in a significant decrease in HRQoL. To assess this relation between patient satisfaction and expectations regarding treatment and HRQoL in more detail, we correlated the CTSQ domains with the HRQoL domains and items. No positive correlations were found between the ET domain and any of the HRQoL domains or items, indicating that not all concepts of the CTSQ are identified by HRQOoL questionnaires. As argued before, expectations of therapy are likely to be influenced by the information patients have received. However, satisfaction seems also to be influenced by patients’ opinions regarding the received information as several studies investigating patient satisfaction reported increased satisfaction when adequate information was provided by healthcare professionals [26–28]. Moreover, satisfaction with information has been associated with better HRQoL [29]. Therefore, we assume the CTSQ may give additional clinically relevant information that is not provided by HRQoL questionnaires regarding patients’ expectations and satisfaction with information provision and possibly also other aspects of cancer care.

Terwee et al. [11] suggested that a sample size of at least 50 patients would be sufficient for a validation study. Nevertheless, for the clinical interpretation of the scores, a larger sample size may be needed to get more reliable results as we were not able to calculate the effect size in the known-groups comparison. For this reason, the small sample size may be considered as a limitation in our study.

We were not able to evaluate test–retest reliability since the questionnaire was only given once after the fourth cycle of chemotherapy. If patients fill in the CTSQ a second time after the first completion, it will be hard to define an appropriate interval between those two completions as we included patients who have a relatively poor prognosis. If the interval between these completions is too short, the difficulty may be that they recall their earlier answers upon filling in the CTSQ for a second time. Moreover, when the interval is too long, patients may have progressed in their disease experiencing more adverse events, which may bias our results. However, we do not expect this to be a major problem as this part has already been validated in the psychometric validation study, showing good results [9].

In conclusion, we were able to support the internal consistency reliability and construct validity of the Dutch version of the CTSQ in patients with lung cancer treated with intravenous chemotherapy. Only a few aspects of HRQoL were significantly correlated to items of the CTSQ, indicating the need of using the CTSQ in studies evaluating satisfaction and expectations of patients on cancer chemotherapy. Since patients with disseminated cancer often have a limited prognosis, considering patients’ motivations and needs is of importance to improve HRQoL. We therefore believe that our results may encourage researchers to use the CTSQ to investigate patients’ expectations and satisfaction with therapy in future studies.
